# Alpha-Amylase and Alpha-Glucosidase Enzyme Inhibition and Antioxidant Potential of 3-Oxolupenal and Katononic Acid Isolated from *Nuxia oppositifolia*

**DOI:** 10.3390/biom10010061

**Published:** 2019-12-30

**Authors:** Ali S. Alqahtani, Syed Hidayathulla, Md Tabish Rehman, Ali A. ElGamal, Shaza Al-Massarani, Valentina Razmovski-Naumovski, Mohammed S. Alqahtani, Rabab A. El Dib, Mohamed F. AlAjmi

**Affiliations:** 1 Medicinal, Aromatic and Poisonous Plants Research Center (MAPRC), College of Pharmacy, King Saud University, PO Box 2457, Riyadh 11451, Saudi Arabia; alalqahtani@ksu.edu.sa (A.S.A.); hidayathsyed@gmail.com (S.H.); 2 Department of Pharmacognosy, College of Pharmacy, King Saud University, PO Box 2457, Riyadh 11451, Saudi Arabia; aelgamel@ksu.edu.sa (A.A.E.); shazamas@yahoo.com (S.A.-M.); malajmii@ksu.edu.sa (M.F.A.); 3 South Western Sydney Clinical School, School of Medicine, University of New South Wales, Sydney, NSW 2052, Australia; v.naumovski@unsw.edu.au; 4 Department of Pharmaceutics, College of Pharmacy, King Saud University, Riyadh 11451, Saudi Arabia; msaalqahtani@ksu.edu.sa; 5 Department of Pharmacognosy, Faculty of Pharmacy, Helwan University, Cairo 11795, Egypt; reldib@yahoo.com

**Keywords:** *Nuxia oppositifolia*, antioxidant, 3-oxolupenal, katononic acid, DPPH, ABTS, glucosidase, amylase, docking

## Abstract

*Nuxia oppositifolia* is traditionally used in diabetes treatment in many Arabian countries; however, scientific evidence is lacking. Hence, the present study explored the antidiabetic and antioxidant activities of the plant extracts and their purified compounds. The methanolic crude extract of *N. oppositifolia *was partitioned using a two-solvent system. The *n*-hexane fraction was purified by silica gel column chromatography to yield several compounds including katononic acid and 3-oxolupenal. Antidiabetic activities were assessed by α-amylase and α-glucosidase enzyme inhibition. Antioxidant capacities were examined by 2,2-diphenyl-1-picrylhydrazyl (DPPH) and 2,2′-azino-bis(3-ethylbenzthiazoline-6-sulfonic acid) (ABTS) scavenging assays. Further, the interaction between enzymes (α-amylase and α-glucosidase) and ligands (3-oxolupenal and katononic acid) was followed by fluorescence quenching and molecular docking studies. 3-oxolupenal and katononic acid showed IC_50_ values of 46.2 μg/mL (101.6 µM) and 52.4 μg/mL (119.3 µM), respectively against the amylase inhibition. 3-oxolupenal (62.3 µg/mL or 141.9 μM) exhibited more potent inhibition against α-glucosidases compared to katononic acid (88.6 µg/mL or 194.8 μM). In terms of antioxidant activity, the relatively polar crude extract and *n*-butanol fraction showed the greatest DPPH and ABTS scavenging activity. However, the antioxidant activities of the purified compounds were in the low to moderate range. Molecular docking studies confirmed that 3-oxolupenal and katononic acid interacted strongly with the active site residues of both α-amylase and α-glucosidase. Fluorescence quenching results also suggest that 3-oxolupenal and katononic acid have a good affinity towards both α-amylase and α-glucosidase enzymes. This study provides preliminary data for the plant’s use in the treatment of type 2 diabetes mellitus.

## 1. Introduction

Type 2 diabetes mellitus (T2DM) is characterized by elevated blood glucose levels and can lead to serious complications such as nephropathy, neuropathy, retinopathy, and cardiovascular disease [1,2]. T2DM remains one of the most common health issues and accounts for 90% of the cases of diabetes, with a mortality incidence of 4.9 million people worldwide [3]. One of the therapeutic approaches of controlling postprandial hyperglycemia in T2DM is inhibiting the digestion of dietary carbohydrates. Pancreatic α-amylase (E.C. 3.2.1.1) is a key enzyme that breaks down dietary carbohydrates such as starch into simple monosaccharides in the digestive system. These are further degraded by α-glucosidases to glucose which, on absorption, enters the bloodstream. Therefore, inhibiting α-amylase and α-glucosidase enzymes can suppress carbohydrate digestion, delay glucose uptake and consequently, reduce blood sugar levels [4]. Although drugs such as acarbose, voglibose, and miglitol inhibit α-glucosidase and α-amylase in practice, they produce undesired side effects such as bloating, abdominal discomfort, diarrhea, and flatulence [5].

In addition, many chronic diseases such as T2DM have been linked to oxidative stress which involves the production of reactive oxygen species (ROS) such as superoxide anion radical (O_2_^−^), hydrogen peroxide (H_2_O_2_) and hydroxyl radical (OH^−^). The role of free radicals in the pathogenesis and progression of diabetes is confirmed by an increase in the concentration of malondialdehyde (MDA) by lipid peroxidation in the pancreatic tissue of diabetic animal models [6]. Thus, compounds that have both antidiabetic and antioxidant activities, without causing serious adverse effects, would be beneficial.

**Figure 1:**
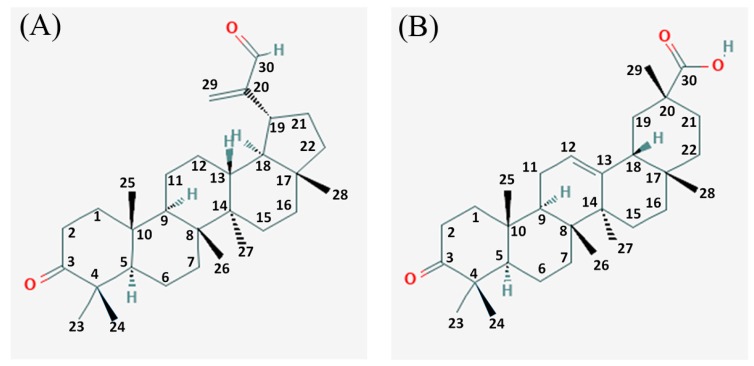
Structures of (**A**) 3-oxolupenal, and (**B**) katononic acid.

The genus *Nuxia* belongs to family *Stilbaceae* and comprises of 40 species of shrubs and trees in the southern region of the Arabian Peninsula, tropical Africa, and South Africa. Two species of *Nuxia,* viz. *N*. *oppositifolia* (Hochst.) Benth and *N. congesta* R. Br. Fresen, are found in Saudi Arabia with limited distribution in the southwestern parts of the Hijaz area [7]. *Nuxia* species are traditionally used to treat various diseases such as malaria, splenomegaly, venereal disease, as purgatives, and in the treatment of infantile hydrocephalus and urine albumin [8]. Some species of *Nuxia* have been traditionally used in the management of type II diabetes mellitus. The infusion of bark and roots are taken orally to control diabetes [9]. The phytochemical analysis of *Nuxia* plants has shown that they are a rich source of phenylpropanoid glycosides such as verbascoside [10], clerodane and labdane diterpenoids and pentacyclic triterpenes [8]. Recently, Al-Massarani et al. (2017) reported the isolation of three new labdane diterpenoic acids, a new seco-triterpene (3,4-seco-olean-12-en-3,30 dioic acid) along with some known triterpenes (3-oxolupenal and katononic acid) and phytosterols such as β-sitosterol and stigmasterol [11]. Many studies have suggested the use of triterpenes in the prevention of diabetic complications such as nephropathy, embryopathy, neuropathy, or impaired wound healing [12]. For instance, oleanolic acid, betulinic acid, and ursolic acid are potent inhibitors of the TGR5 receptor, which is involved in energy metabolism in brown adipose tissue [13,14,15]. The antioxidant potential of ursolic acid (hydroxyl radical and superoxide anions scavenging activity; inhibits activation of receptor for advanced glycation end products (RAGE)-NADPH oxidase-NF-κB signal pathway), corosolic acid (reduces levels of thiobarbituric acid-reactive substance and 8-hydroxydeoxyguanosine, which are oxidative stress biomarkers), arjunoic acid (inhibits STZ-induced intracellular levels of reactive nitrogen species (RNS) and reactive oxygen species (ROS) in spleen; deactivates polyol pathway; enhances IL-2 and IFN-γ levels and decreases TNF-α levels) and bacosine (decreases malonylaldehyde level; increases glutathione level; enhances superoxide dismutase and catalase activities in liver) is well documented [16,17,18,19,20]. Until now, there are no studies of the antidiabetic effects of 3-oxolupenal and katononic acid terpenoids. Therefore, this study evaluated the antidiabetic and antioxidant potential of *N. oppositifolia* extracts and compounds (3-oxolupenal and katononic acid). The 2-dimensional structures of 3-oxolupenal and katononic acid are shown in Figure 1.

## 2. Materials and Methods

### 2.1. Chemicals and Reagents

Porcine α-amylase, α-glucosidase, diphenyl-1-picrylhydrazyl (DPPH), butylated hydroxytoluene (BHT), 2,2′-azino-bis(3-ethylbenzothiazoline-6-sulfonic acid) diammonium salt (ABTS), *p*-Nitrophenyl-α-glucopyranoside (PNPG) and acarbose were purchased from Sigma-Aldrich Co., St Louis, MO, USA, while soluble starch (extra pure) was obtained from HiMedia Laboratories (Mumbai, India). Other chemicals and reagents were of analytical grade. Water was double distilled.

### 2.2. Plant Material

The aerial parts of *N. oppositifolia* (leaves, stems, and flowers) were collected in March 2012 from Wadi Lajab, Jazan province, Saudi Arabia. The voucher specimen (Voucher # 15501) was deposited at the Pharmacognosy Department, College of Pharmacy, King Saud University after identification by a taxonomist.

#### Extraction and Isolation

The aerial parts of *N. oppositifolia* (900 g) were dried, powdered, and then extracted three times with 80% methanol as described earlier [11]. The extracts were filtered and concentrated under reduced pressure at 40 °C using a rotary vacuum evaporator (Buchi, New Castle, PA, USA). The dried crude extract (105 g) was re-dissolved in 40% methanol, and subjected to sequential liquid-liquid extraction with a solvent series: *n*-hexane, chloroform, and *n*-butanol. Each fraction was weighed individually after complete evaporation to dryness and stored at 4 °C until further use. A portion of the crude extracts was stored for further studies.

### 2.3. Isolation and Identification of 3-Oxolupenal and Katononic Acid

Each fraction was examined for its inhibitory activity against α-amylase and α-glucosidase enzymes. The *n*-hexane fraction (17.6 g) showed maximum activity, and was further fractionated using a pre-packed silica gel column (40 mm i.d. × 350 mm) and eluted with *n*-hexane-ethyl acetate gradient, following the method described in Al-Massarani et al. (2017) [11]. The collected fractions were examined with thin-layer chromatography (TLC) and similar fractions were pooled together into four different sub-fractions (A–D). The sub-fractions were tested for α-amylase and α-glucosidase inhibitory activity; the sub-fraction (A and B) which showed the highest inhibition was further analyzed. Sub-fraction A which was eluted with 5% EtOAc/*n*-hexane, was further purified using a Chromatotron device) (San Bruno, CA, USA) (0.5% EtOAc/*n*-hexane) to yield 3-oxolupenal (170 mg), as described by Al-Massarani et al. (2017) [11]. Similarly, sub-fraction B which was eluted with 10% EtOAc/*n*-hexane gave katononic acid (153 mg) upon crystallization.

The isolated compounds namely 3-oxolupenal and katononic acid were characterized using GC-MS, ^13^C, and ^1^H NMR (Supplementary Figures S1–S3). The GC-MS analyses were performed using a Perkin Elmer Clarus 600 T gas chromatograph) (Shelton, CT, USA) connected to a Turbomass spectrometer at the Research Center, College of Pharmacy, King Saud University. An aliquot of 1 µL of oil was injected into the Elite-5MS column of 30 m, 0.25 µm film thickness, and 0.25 mm internal diameters. The initial oven temperature of the GC-MS system was kept at 40 °C for 2 min, then increased to 150 °C at a rate of 2.5 °C/min, and finally to 300 °C at a rate of 1 °C/min. The injector temperature was maintained at 280 °C. The interface temperature was 250 °C. Helium was used as a mobile phase at a flow rate of 1.0 mL/min. Mass spectral detection was carried out in electron ionization mode by scanning at 40 to 600 (*m*/*z*). Finally, the unknown compounds were identified by comparing the spectra with that of the National Institute of Standard and Technology library. The total time required for analyzing a single sample was 60 min.

### 2.4. Inhibition Assay for Amylase Activity

The inhibition of amylase activity was carried out by following the method described by Young et al. (2008) [21]. The different fractions of the plant and compounds were prepared using 5% dimethyl sulfoxide (DMSO). Fraction/compound (500 µL) of 100 µg/mL concentration (i.e., 50 µg) and 500 µL of 20 mM sodium phosphate buffer (pH 6.8) containing 20 µL of amylase (0.5 mg/mL) were incubated at 25 °C for 10 min. After pre-incubation, 500 µL of 1% starch solution in 0.02 M sodium phosphate buffer (pH 6.9) were added to each tube and incubated for 15 min. The reaction was stopped with 1.0 mL of dinitrosalicyclic acid. The test tubes were then incubated in a boiling water bath for 5 min and cooled to room temperature. The reaction mixture was then diluted after adding 10 mL distilled water, and the absorbance (Abs) was measured at 540 nm. Mean values were obtained from triplicate experiments. The control samples were prepared without any plant extracts/fractions/compounds. The percent inhibition of amylase activity was calculated using the following formula:

(1) $$Inhibition(\%)=\left(\frac{Abs_{Control}-Abs_{Compound}}{Abs_{Control}}\right)\times 100$$

The mean values were obtained from triplicate experiments.

### 2.5. Inhibition Assay for Glucosidase Activity

Glucosidase inhibition assay was carried out by following the method of Elya et al. (2008) [22]. The different fractions of the plant and compounds were prepared using 5% dimethyl sulfoxide (DMSO). Phosphate buffer (100 mM, pH 6.8; 1 mL) and 80 µL of the fraction/compound of 100 µg/mL (i.e., 8 µg) concentration were mixed with 20 μL of α-glucosidase (0.01 mg/mL) and incubated at 37 °C for 10 min. Next, 50 μL of 5 mM p-nitrophenyl-α-D-glucopyranoside (pNPG) were added to the mixture to start the reaction. The reaction mixture was incubated at 37 °C for 60 min and stopped by adding 2.5 mL of 0.1 M Na_2_CO_3._ The α-glucosidase activity was determined by measuring the absorbance (Abs) at 400 nm. The mean values were obtained from triplicate experiments. The inhibition of glucosidase activity was calculated as percentage inhibition using Equation (1).

### 2.6. DPPH Radical Scavenging Activity

The DPPH radical scavenging method measured the antioxidant activity following the method of Braca et al. (2001) with slight modification [23]. The crude extract, fractions, and compounds at 100 µg/mL concentrations were added to 3 mL of 0.004% DPPH solution. In the control sample, methanol was added in place of test extract/fractions/compounds. The DPPH is violet in color at room temperature and the color changes to yellow by either the process of hydrogen or electron donation by antioxidants. Absorbance was measured at 520 nm after 30 min incubation. The percent inhibition was calculated using Equation (1).

### 2.7. ABTS Radical Cation Scavenging Activity

The ABTS radical scavenging activity of the samples was measured using the method of Re et al. (1999) with slight modification [24]. ABTS solution in water (7 mM concentration) and an aqueous solution of potassium persulfate (2.45 mM) were prepared. The two solutions were mixed in the volume ratio 1:1 and stored in the dark 6 h at room temperature to produce the ABTS radical. The blue chromophore, which has a characteristic absorbance at 734 nm, was generated by reacting equal amount of ABTS and potassium persulfate solution. The ABTS stock solution was diluted with ethanol to an absorbance of 0.70 ± 0.02 at 734 nm and equilibrated at 30 °C. The crude extract, different fractions and isolated compounds at 100 µg/mL concentrations were mixed with 2.9 mL of diluted ABTS radical cation solution. The reaction was incubated at 30 °C for 20 min, and the absorbance at 734 nm was measured. The affinity of the test sample to quench the ABTS free radical was evaluated using Equation (1).

### 2.8. Fluorescence Quenching-Based Binding Studies

Quenching in the intrinsic fluorescence of proteins (α-amylase and α-glucosidase) was observed in the absence and presence of quenchers (3-oxolupenal and katononic acid) in 100 mM sodium phosphate buffer (pH 6.8) at 298 K as reported earlier [25]. Proteins were excited at 295 nm and the fluorescence spectra were recorded in the 300–450 nm range. The excitation and emission slit widths were set at 5 nm each. The concentration of proteins was fixed at 2 µM while the concentration of quenchers was varied. The inner filter effect contributed by quenchers was corrected using the following relation [26].

(2) $$F_{corr}=F_{obs}\times e^{\left(A_{ex}+A_{em}\right)/2}$$

where *F_corr_* is the corrected fluorescence intensity, *F_obs_* is the observed fluorescence intensity, *A_ex_* is the absorbance at the excitation wavelength, and *A_em_* is the absorbance at the emission wavelength.

### 2.9. Molecular Docking Studies

The preparation of the proteins/ligands, generation of receptor grid, and docking were performed on AutoDock 4.2 as described recently [27]. The SDF files of 3-oxolupenal and katononic acid were retrieved from PubChem database bearing PubChem CIDs 11,848,142 and 9,981,416, respectively. The energies of the ligands were minimized using Universal forcefield (UFF) in OpebBabel. Gasteiger partial charges were added, non-polar hydrogen atoms were merged and rotatable bonds were defined. The crystal structure of α-amylase (PDB Id: 4GQR; resolution 1.2 Å) [28] and α-glucosidase (PDB Id: 5NN5; resolution 2.0 Å) [29] were downloaded from the PDB database (http://www.rcsb.org/pdb). AutoDock tool (ADT) was employed to prepare proteins by adding missing hydrogen atoms, assigning Kollman united atom type charges and solvation parameters at pH 7.4 to mimic the physiological environment. Grid maps of 50 × 70 × 60 Å and 80 × 80 × 80 Å dimensions with 0.375 Å spacing were prepared using AutoGrid. Other AutoDock parameters were set at their default values. Molecular docking employed the Lamarck Genetic Algorithm (LGA) and the Solis and Wets search methods. A total of 2,500,000 energy calculations were performed for each run and a total 10 calculations were performed. All the other parameters were set at their default values. The docking procedure implemented in this study was authenticated by re-docking the ligands present in the X-ray structure files of α-amylase and α-glucosidase and comparing the RMSDs between the docked pose and X-ray pose.

### 2.10. Statistical Analysis

Results are expressed as means of three independent experiments ± standard deviations. Statistical difference between the compounds was tested by one-way analysis of variance (ANOVA) using the GraphPad (version 6.1, San Diego, CA, USA), and individual comparisons were obtained by Duncan’s new multiple range test. A difference in the mean values of (*p* < 0.05) was considered statistically significant.

## 3. Results and Discussion

### 3.1. α-Amylase and α-Glucosidase Inhibition Studies

It has been reported that the activity of human pancreatic α-amylase (HPA) in the small intestine correlates to an increase in post-prandial glucose levels, the control of which is, therefore, an important aspect in the treatment of T2DM [30]. Among the fractions, *n*-hexane fraction exhibited significant amylase inhibition compared to other fractions at 100 µg/mL. The lowest inhibition against amylase was observed in the chloroform fraction. Debasis et al. (2013) had observed the highest α-amylase and α-glucosidase inhibitory activity in the *n*-hexane fraction of sepal *of Salmalia malabarica* [31]. The *n*-hexane fraction of *Myagropsis myagroides* showed significant amylase inhibition [32]. Saleh et al. (2013) observed significant α-glucosidase and α-amylase inhibitory in the *n*-hexane fractions of ten plant extracts traditionally used in Iran for diabetes [33,34].

**Figure 2:**
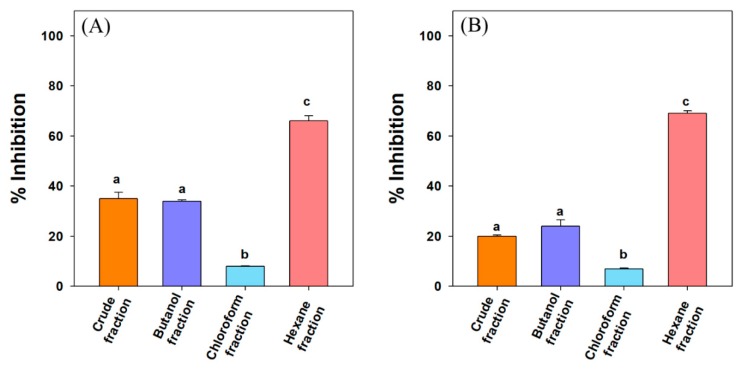
Inhibitory effect of *N. oppositifolia* crude extract and different fractions against (**A**) pancreatic α-amylase, and (**B**) pancreatic α-glucosidase at 100 µg/mL concentration. Data are presented as mean ± standard deviation values of triplicate determinations. Different superscripts letters (a–c) for a given value within the figure are significantly different from each other (Duncan’s multiple range test (*p* < 0.05).

Our result shows that the *n*-hexane fraction contained the compounds responsible for α-amylase activity inhibition hence, further purification of the extract was carried out. The sub-fractions with the highest inhibitory activity were characterized by GC-MS (Figure S1) and ^1^H/^13^C NMR data (Figures S2 and S3), which confirmed the presence of 3-oxolupenal and katononic acid. Many reports have related the α-amylase inhibitory action of the extracts to terpenoids present in the extracts [35,36,37]. The order of inhibition for the crude extract and different fractions at 100 µg/mL was *n*-hexane fraction (66%) > crude extract (35%) > *n*-butanol fraction (34%) > chloroform fraction (8%) (Figure 2A). The α-amylase inhibitory potential (IC_50_ value) of the pure compounds isolated from the *n*-hexane fraction follows the order of acarbose (27.3 µg/mL; 42.3 µM) > 3-oxolupenal (46.2 µg/mL; 105.3 µM) > katononic acid (52.4 µg/mL; 115.2 µM) (Table 1). From the results, it was clear that katononic acid and 3-oxolupenal inhibited α-amylase enzyme significantly, and their efficacy was comparable to the standard acarbose. Previous studies have shown α-amylase inhibitory activity by various terpenes such as ursolic, corosolic, and oleanolic acids. The IC_50_ values of these terpenes were in the range of 22.6–94.1 μM, which correlates to the present study [38]. Similarly, Zhang et al. (2017) observed significant inhibition of α-amylase by pentacyclic triterpenes consisting of the ursane skeleton [38]. The presence of the ursane ring in 3-oxolupenal and katononic acid might be responsible for the observed anti-amylase activity. Previously, it has been observed that the anti-amylase activity of ursolic acid was greater than that of oleanolic acid, suggesting that the C-29 methyl group at C-19 in ursolic acid instead of C-20 in oleanolic acid played a significant role in inhibiting amylase.

The final step of carbohydrate hydrolysis to produce absorbable monosaccharide is mediated by the α-glucosidases enzymes in epithelium tissue of the small intestine. The inhibition of these enzymes slows down the breakdown of dietary polysaccharides into the simpler saccharides in the gastro-intestinal tract, thus reducing postprandial hyperglycemia [39]. In the present study, the order of α-glucosidase inhibition for the different fractions was *n*-hexane fraction (69%) > *n*-butanol fraction (24%) > crude extract (20%) > chloroform fraction (7%) (Figure 2B). The isolated compound 3-oxolupenal showed significantly (*p >* 0.05) higher α-glucosidase inhibition activity, with an IC_50_ value of 62.2 µg/mL (141.8 μM) compared to katononic acid 88.6 µg/mL (194.8 μM) (Table 1). The anti-α-glucosidase activity of the compounds isolated from the *n*-hexane fraction was 2-3 fold less than the standard acarbose (38.1 µg/mL; 59.0 µM). Many terpenoids such as betulinic, ursolic, corosolic, oleanolic acids, as well as betulin, have been reported as inhibitors of α-glucosidase, with IC_50_ values ranging between 12.1–35.6 μM. However, the inhibitory potential of katononic acid differed significantly compared to 3-oxolupenal, and this may have been due to the poor solubility of triterpenoids in the reaction solutions, which is consistent with previous findings [40]. As for α-amylase, many studies of α-glucosidase inhibitory activity of plant extracts have linked the effect to their terpenoid content [41].

### 3.2. DPPH and ABTS Scavenging Activity

**Figure 3:**
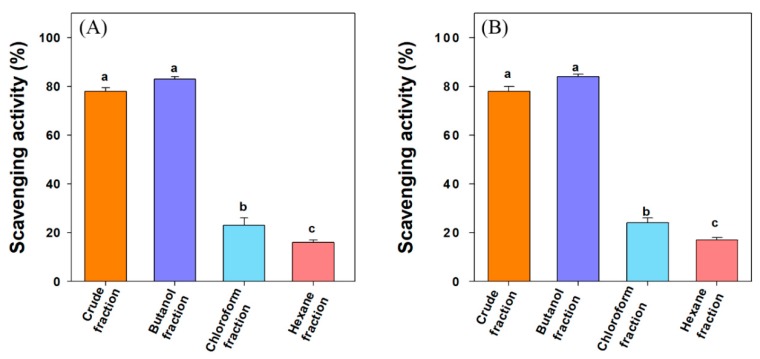
Comparison of (**A**) 2,2-diphenyl-1-picrylhydrazyl (DPPH) scavenging activity, and (**B**) 2,2′-azino-bis(3-ethylbenzthiazoline-6-sulfonic acid) (ABTS) scavenging activity of *N. oppositifolia* crude extract and different fractions at 100 µg/mL concentration. Data are presented as mean ± standard deviation values of triplicate determinations. Different superscripts letters (a–c) for a given value within the figure are significantly different from each other using Duncan’s multiple range test (*p* < 0.05).

Free radical-mediated oxidative stress is believed to be the primary cause of many disorders such as diabetes mellitus, arthritis, cancer, cardiovascular diseases, and aging. Recent reports show an increase in the concentration of free radicals in T2DM [42]. Figure 3A shows the DPPH radical scavenging activity of crude extract and different fractions at 100 µg/mL concentration. The order of the DPPH scavenging activity was thus *n*-butanol fraction (83%) > crude fraction (78%) > chloroform fraction (23%) > *n*-hexane fraction (16%). The higher antioxidant activities of butanol and crude fractions may be attributed to the presence of many polyphenols. The compounds isolated from *n*-hexane fraction i.e., 3-oxolupenal and katononic acid, showed modest antioxidant activities in terms of their IC_50_ values of 188.5 µM and 192.4 µM, respectively (Table 2).

Many reports suggest the use of multiple methods to evaluate the antioxidant potential of compounds as the DPPH assay has certain disadvantages, such as partial ionization of the tested compounds and pH dependence [43]. For comparison, the antioxidant activities of 3-oxolupenal and katononic acid were also evaluated by ABTS scavenging activity. All the fractions of *N. oppositifolia* demonstrated an appreciable ABTS radical scavenging activity. The ABTS radical scavenging activity was in the range of 17–84% at 100 µg/mL concentration of crude extract and different fractions. The order of the ABTS scavenging activity was *n*-butanol fraction (84%) > crude extract (78%) > chloroform fraction (24%) > *n*-hexane fraction (17%) (Figure 3B). The higher antioxidant activities of butanol and crude fractions may be attributed to the presence of many polyphenols. For 3-oxolupenal and katononic acid, the IC_50_ values of ATBS scavenging activity were estimated to be 191.9 µM and 181.9 µM, respectively (Table 2). Adewusi et al. (2011) noted the IC_50_ value of 230 µg/mL for DPPH and 140 µg/mL in ABTS scavenging assay for *Buddleja salvifolia* methanolic extract, which belongs to the same family as the *Nuxia* species [44]. Amoo et al. (2009) observed 96% DPPH scavenging activity at 100 µg/mL of a 50% aqueous methanolic extract of *Buddleja salviifolia* [45]. Similar results have been observed in the previous studies carried out by Gangwar et al. (2014) [46].

### 3.3. Interaction between 3-Oxolupenal and Katononic Acid with α-Amylase and α-Glucosidase

**Figure 4:**
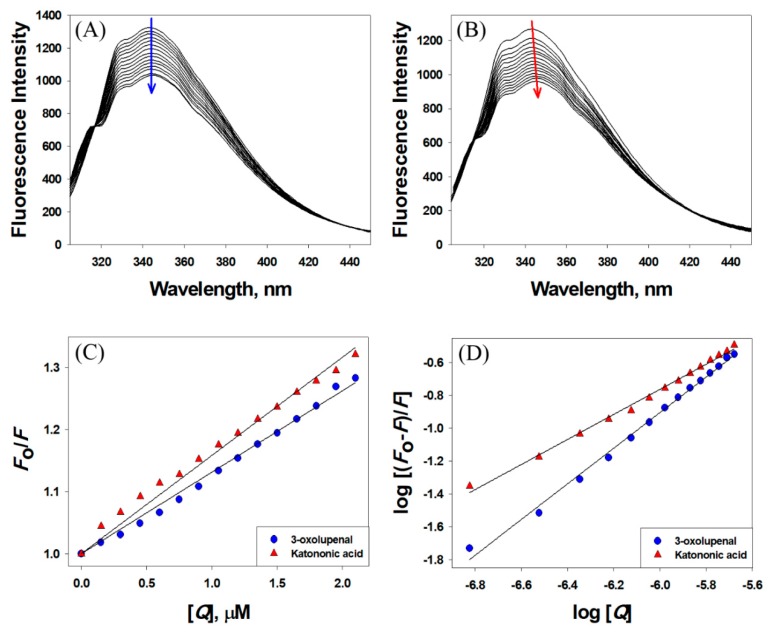
Interaction of α-amylase with 3-oxolupenal and katononic acid. Quenching in the fluorescence intensity of α-amylase in the presence of (**A**) 3-oxolupenal and (**B**) katononic acid. The binding parameters were deduced from (**C**) Stern–Volmer and (**D**) modified Stern–Volmer plots.

**Figure 5:**
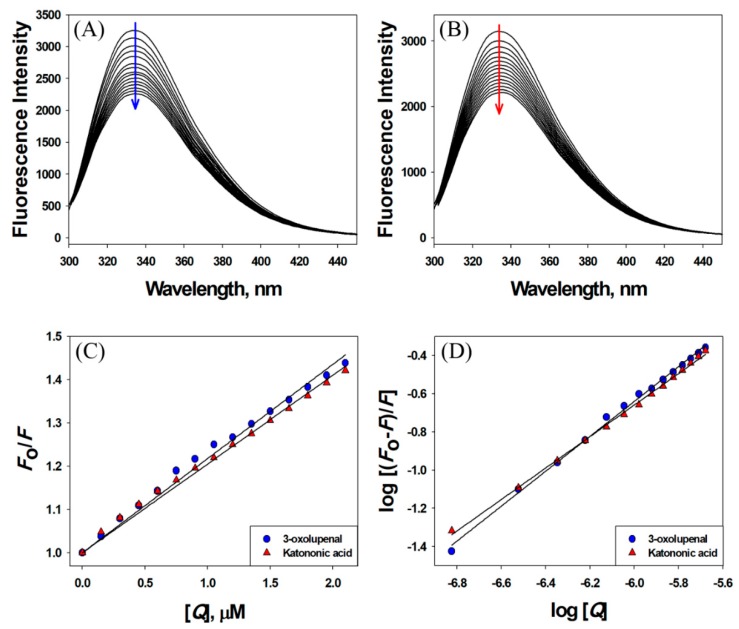
Interaction of α-glucosidase with 3-oxolupenal and katononic acid. Quenching in the fluorescence intensity of α-glucosidase in the presence of (**A**) 3-oxolupenal and (**B**) katononic acid. The binding parameters were deduced from (**C**) Stern–Volmer and (**D**) modified Stern–Volmer plots.

Observing quenching in the fluorescence intensity of a protein in the presence of a ligand is a powerful tool in determining the binding parameters such as binding affinity and Gibb’s free energy of stabilization [47]. Here, we have followed quenching in the fluorescence intensity of α-amylase and α-glucosidase in the presence of 3-oxolupenal and katononic acid at 298 K (Figure 4 and Figure 5). The results showed a decrease in the fluorescence intensities of α-amylase and α-glucosidase in the presence of 3-oxolupenal and katononic acid, thus suggesting an interaction of 3-oxolupenal and katononic acid with α-amylase and α-glucosidase (Figure 4A,B and Figure 5A,B). The binding parameters for the interaction were deduced using the following Stern–Volmer and modified Stern–Volmer equations.

(3) $$\frac{F_{o}}{F}=1+K_{SV}[Q]=1+k_{q}\tau_{o}[Q]$$

(4) $$\log\left(\frac{F_{o}-F}{F}\right)=\log K_{a}+n\log[Q]$$

where, *F_o_* and *F* are the fluorescence intensities of enzymes (α-amylase and α-glucosidase) in the absence and presence of ligands (3-oxolupenal and katononic acid); *K_SV_* is the Stern–Volmer constant; [*Q*] is the molar concentration of ligands (3-oxolupenal and katononic acid); *k_q_* is the bimolecular quenching rate constant; *τ_o_* is the lifetime of enzymes’ fluorescence in the absence of ligands (=5.71 × 10^−9^ s [48]); *K_a_* is the binding constant; *n* is the number of binding sites on the enzyme.

The results presented in Table 3 show that the Stern–Volmer quenching constant of 3-oxolupenal and katononic acid were of the order of 10^5^ M^−1^, indicating strong quenching in the fluorescence of enzymes (α-amylase and α-glucosidase) by 3-oxolupenal and katononic acid (Figure 4C and Figure 5C). Further, the bimolecular quenching rate constants for the interaction between ligands (3-oxolupenal and katononic acid) and enzymes (α-amylase and α-glucosidase) has been found to be of the order of 10^13^ M^−1^ s^−1^, which are at least 1000 times higher than the diffusion rate constant (=10^10^ M^−1^ s^−1^) [49]. These results suggest that the observed quenching in the fluorescence of enzymes was due to the formation of a complex between enzymes and ligands i.e., through static quenching mechanism. Furthermore, the analysis of the modified Stern–Volmer equation shows that the binding constants of enzymes towards ligands were of the order of 10^4^–10^5^ M^−1^, indicating a moderate to high binding affinity. Similarly, the number of binding sites for the ligands were estimated to be around one per molecule of enzymes (Table 3). We found that 3-oxolupenal binds to the active site of α-amylase and α-glucosidase more efficiently as compared to katononic acid. These results support our observations that 3-oxolupenal had higher anti-amylase and anti-glucosidase activities as compared to katononic acid.

### 3.4. Molecular Docking Studies

#### 3.4.1. Authentication of Molecular Docking Method

The molecular docking procedures were authenticated by predicting the binding pose of ligands in the protein–ligand complex crystal structures of the respective proteins. For this, the ligand present in an X-ray crystal structure was extracted and re-docked into the binding site of the protein. The pose of the ligand in the X-ray structure was compared with the docked pose and root mean square deviation (RMSD) was calculated. We found that the RMSDs between the X-ray poses and docked poses of myricetin and 1-deoxynojirimycin at the respective active sites of α-amylase and α-glucosidase were 0.3849 Å and 0.8936 Å, respectively (Supplementary Figure S4). As the RMSDs of the ligand between X-ray and docked poses were significantly lower than the maximum allowed value of 2.0 Å, this indicated that the docking procedure was valid.

#### 3.4.2. Molecular Docking of 3-Oxolupenal and Katononic Acid with α-Amylase

**Figure 6:**
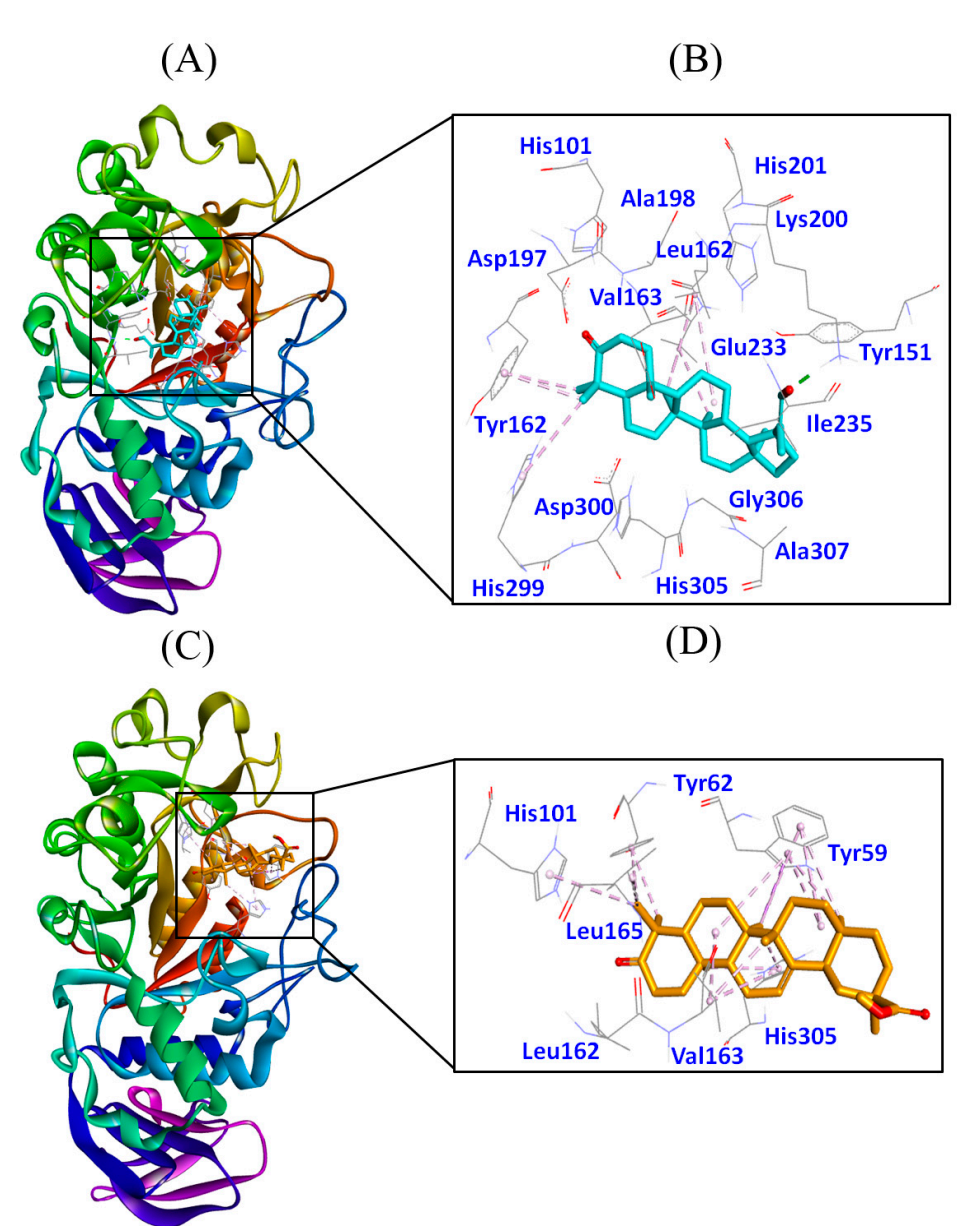
Molecular docking of 3-oxolupenal and katononic acid with α-amylase. (**A**) Binding of 3-oxolupenal with α-amylase, (**B**) amino acid residues and various interactions involved in 3-oxolupenal-α-amylase complex, (**C**) binding of katononic acid with α-amylase, and (**D**) amino acid residues and various interactions involved in katononic acid-α-amylase complex.

We have performed molecular docking of 3-oxolupenal and katononic acid with α-amylase, and the results are presented in Figure 6, and Table 4. 3-oxolupenal sits at the active site of α-amylase and the complex was stabilized by one hydrogen bond and seven hydrophobic interactions (Figure 6A,B and Table 4). A hydrogen bond was formed between the HZ2-atom of Lys200 and the O-atom of the ligand. Similarly, Leu162, Val163, Tyr62, and His299 residues were involved in making hydrophobic interactions (Table 4). Other charged and polar residues such as His101, Tyr151, Asp197, Ala198, His201, Glu233, Ile235, His299, Asp300, His305, Gly306, and Ala307 were involved in making van der Waal’s interactions (Figure 6B). Similarly, katononic acid was found to bind strongly to the catalytic site of α-amylase, and the complex was stabilized with sixteen hydrophobic interactions (Figure 6C,D and Table 4). The residues involved in making hydrophobic interactions were Trp59, Tyr62, His101, Val163, Leu165, and His305. In addition, Leu162 interacted with katononic acid through van der Waal’s interaction (Figure 6D). The Gibb’s free energy for the α-amylase-3-oxolupenal complex was predicted to be −9.1 kcal/mol, corresponding to a binding affinity of 4.72 × 10^6^ M^−1^. Similarly, Gibb’s free energy for the α-amylase-katononic acid complex was found to be −8.6 kcal/mol, corresponding to a binding affinity of 2.03 × 10^6^ M^−1^. The magnitude of the binding energy and binding affinity indicates that 3-oxolupenal and katononic acid interacted strongly with α-amylase, confirming the in vitro data.

The X-ray crystal structure and enzyme kinetics studies have revealed that the active site of α-amylase is characterized by the presence of three important residues namely Asp197, Glu233, and Asp300 [28,50]. In the hydrolysis of polymeric substrates such as starch, Asp197 acts as a nucleophile, while Glu233 acts in acid–base catalysis during the hydrolysis reaction and Asp300 acts as a key residue in optimizing the orientation of the substrate [28,50]. Moreover, Williams et al. (2012) found that myricetin (an inhibitor of α-amylase) formed four hydrogen bonds with Gln63, His101, and Asp197 and two hydrophobic interactions with Tyr62 and Leu165. Other amino acid residues of α-amylase interacting with myricetin were Trp59, Val98, Leu162, Val163, Ala198, and Glu233 [28]. Our docking results confirmed that 3-oxolupenal and katononic acid interact with several key amino acid residues of α-amylase, which were involved in α-amylase-myricetin complex formation. Further, our in vitro anti-α-amylase activity also confirmed that 3-oxolupenal and katononic acid act as potential inhibitors of the α-amylase enzyme.

#### 3.4.3. Molecular Docking of 3-Oxolupenal and Katononic Acid with α-Glucosidase

**Figure 7:**
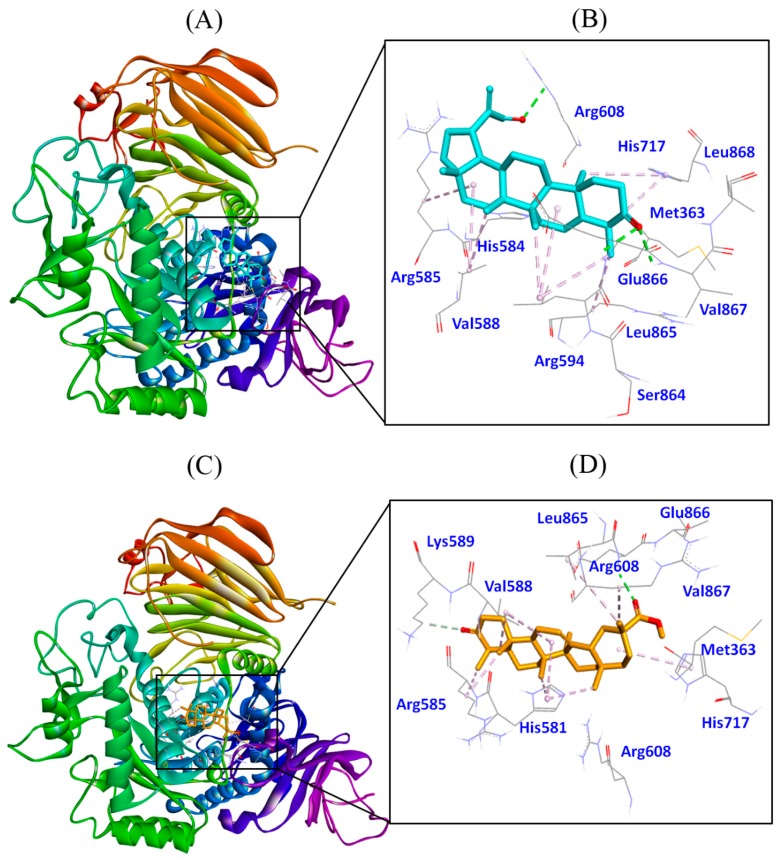
Molecular docking of 3-oxolupenal and katononic acid with α-glucosidase. (**A**) Binding of 3-oxolupenal with α-glucosidase, (**B**) amino acid residues and various interactions involved in 3-oxolupenal-α-glucosidase complex, (**C**) binding of katononic acid with α-glucosidase, and (**D**) amino acid residues and various interactions involved in katononic acid-α-glucosidase complex.

The results showed that both 3-oxolupenal and katononic acid were able to bind to the active site of α-glucosidase (Figure 7 and Table 4). In terms of binding energy and binding affinity, the docking results specified that 3-oxolupenal and katononic acid interacted strongly with α-glucosidase. The Gibb’s free energy of binding was found to be −7.9 kcal/mol, corresponding to a binding affinity of 6.22 × 10^5^ M^−1^ for 3-oxolupenal, and −7.7 kcal/mol corresponding to a binding affinity of 4.44 × 10^5^ M^−1^ for katononic acid. The docking complex of 3-oxolupenal with α-glucosidase was stabilized by three hydrogen bonds and nine hydrophobic interactions (Figure 7A,B and Table 4). The hydrogen bonding residues were Arg608, Glu866, and Val867, while the residues involved in making hydrophobic interactions were Arg585, Val588, Arg594, His717, and Leu865. Other residues such as Met363, His584, Ser864, and Leu868 were also involved in the interaction. Similarly, katononic acid-α-glucosidase complex was stabilized by two hydrogen bonds and ten hydrophobic interactions (Figure 7C,D and Table 4). The residues involved in hydrogen bonding were Lys589 and Glu866, while the residues making hydrophobic interactions were His584, Arg585, Val588, Arg594, His717, and Leu865. Amino acid residues such as Met363, Arg608, and Val867 also contributed to stabilizing the α-glucosidase-katononic acid complex.

Recently, Roig-Zamboni et al. (2017) provided the X-ray crystal structure of human lysosomal acid α-glucosidase with 1-deoxynojirimycin and its derivative N-hydroxyethyl-deoxynojirimycin [29]. A detailed analysis of the X-ray crystal structure of α-glucosidase shows that its active site is located deep in the catalytic GH31 domain [29]. The active site residues such as Asp518 and Asp616 act as a catalytic nucleophile and acid/base donors, respectively, in the classical Koshland double displacement reaction mechanism [51]. 1-Deoxynojirimycin is a well-known amino-sugar inhibitor of α-glucosidase. It binds the active site of α-glucosidase, and the complex is stabilized by a network of hydrogen bonds (Asp404, Asp518, Arg600, Asp616, and His674) and hydrophobic interactions (Trp376, Ile441, Tp516, Met519, Trp613, and Phe649). Other amino acid residues of α-glucosidase that interact with 1-deoxynojirimycin are Leu405, Trp481, Asp645, and Arg672. In the present study, 3-oxolupenal and katononic acid were established to bind at the active site of α-glucosidase at a place close to the binding site of 1-deoxynojirimycin. The results of molecular docking are in agreement with the in vitro anti-α-glucosidase activities of 3-oxolupenal and katononic.

## 4. Conclusions

For many centuries, *N. oppositifolia* has been used by folks in Arabian countries for the management of diabetes. However, in recent times, the full potential of *N. oppositifolia* has not been utilized due to the lack of scientific proof. Previously, our group has isolated various bioactive compounds from the methanolic extract of aerial parts of *N. oppositifolia* [11]. In this study, we performed an activity-guided screening and isolation of bioactive compounds from different fractions of *N. oppositifolia*. The inhibition of α-amylase and α-glucosidase enzymes by different fractions of crude extract has led to the identification of two bioactive compounds, namely 3-oxolupenal and katononic acid. Both compounds (3-oxolupenal and katononic acid) have been isolated from the *n*-hexane fraction of methanolic crude extract of aerial parts of *N. oppositifolia*. The molecular characterization of 3-oxolupenal and katononic acid by GC-MS, ^1^H-NMR, and ^13^C-NMR confirmed their identities. In addition to the inhibition of α-amylase and α-glucosidase, 3-oxolupenal and katononic acid have been found to possess antioxidant activities as evident by DPPH and ABTS scavenging assays. Further, quenching in the fluorescence intensities of α-amylase and α-glucosidase as a consequence of adding 3-oxolupenal and katononic acid confirms a strong interaction between them. Furthermore, molecular docking results confirm the binding of 3-oxolupenal and katononic acid to the active sites of α-amylase and α-glucosidase, thereby explaining the mechanism of enzyme inhibition by these compounds. In summary, the present study suggests enzyme inhibition and antioxidant activity as potential pathways of *N. oppositifolia* in the treatment of diabetes. Since, this is a preliminary study, further mechanistic and *in-vivo* studies should be pursued to confirm the above results.

## Figures and Tables

**Table 1:** Inhibition of pancreatic α-amylase and α-glucosidase activities (IC_50_ values) by different fractions and purified compounds (katononic acid and 3-oxolupenal). Values are means of three experiments ± SD. The values followed by different superscript (a–e) differ significantly (*p* < 0.05).

Fractions/Compounds	Inhibition of α-Amylase (µg/mL)	Inhibition of α-Glucosidase (µg/mL)
Crude extract	90.3 ± 3.2 ^a^	98.4 ± 5.2 ^e^
*n*-butanol fraction	ND	ND
Chloroform fraction	ND	ND
*n*-hexane fraction	73.4 ± 7.3 ^b^	80.3 ± 8.9 ^b^
3-oxolupenal	46.2 ± 5.2 ^c^ (105.3 µM)	62.3 ± 2.4 ^c^ (141.8 µM)
Katononic acid	52.4 ± 3.7 ^c^ (115.2 µM)	88.6 ± 6.2 ^a^ (194.8 µM)
Acarbose (control)	27.3 ± 7.3 ^d^ (42.3 µM)	38.1 ± 3.1 ^d^ (59.0 µM)

ND: Not determined.

**Table 2:** Comparison of DPPH and ABTS scavenging activity (IC_50_ value) of different fractions and purified compounds (3-oxolupenal and katononic acid). Values are means of three experiments ± SD. The values followed by different superscript (a–d) differ significantly (*p* < 0.05).

Fractions/Compounds	DPPH Scavenging Activity (µg/mL)	ABTS Scavenging Activity (µg/mL)
Crude extract	62.3 ± 5.3 ^a^	66.8 ± 8.3 ^a^
*n*-butanol fraction	47.6 ± 4.7 ^b^	43.1 ± 5.2 ^b^
Chloroform fraction	NA	NA
*n*-hexane fraction	NA	NA
3-oxolupenal	82.7 ± 6.7 ^c^ (188.5 µM)	84.2 ± 5.1 ^c^ (191.9 µM)
Katononic acid	87.5 ± 5.4 ^c^ (192.4 µM)	82.7 ± 4.7 ^c^ (181.9 µM)
BHT (control)	16.6 ± 6.2 ^d^ (75.3 µM)	28.0 ± 1.9 ^d^ (127.2 µM)

NA: No activity was observed; BHT: Butylated hydroxytoluene.

**Table 3:** Binding parameters of α-amylase and α-glucosidase with 3-oxolupenal and katononic acid.

Compounds	Stern–Volmer Constant (*K_SV_*) M^−1^	Bimolecular Quenching Rate Constant (*k_q_*) M^−1^ s^−1^	Number of Binding Sites n	Binding Affinity (*K_b_*) M^−1^
**α-Amylase**				
3-oxolupenal	1.31 × 10^5^	2.29 × 10^13^	1.0865	4.11 × 10^5^
Katononic Acid	1.58 × 10^5^	2.77 × 10^13^	0.7646	0.67 × 10^4^
**α-Glucosidase**				
3-oxolupenal	2.18 × 10^5^	3.82 × 10^13^	0.9133	0.69 × 10^5^
Katononic Acid	2.05 × 10^5^	3.59 × 10^13^	0.8239	1.92 × 10^4^

**Table 4:** Molecular docking parameters for 3-oxolupenal and katononic acid interaction with α-amylase and α-glucosidase.

Compounds	Interacting Residues	Type of Interaction	Distance (Å)	Binding Energy, kcal/mol
**α-Amylase**				
3-oxolupenal	Lys200:HZ2-Lig:O	Hydrogen Bond	1.8982	−9.1
	Leu162-Lig	Hydrophobic (Alkyl)	5.3493	
	Lig:C-Val163	Hydrophobic (Alkyl)	4.5101	
	Lig:C-Leu162	Hydrophobic (Alkyl)	5.1043	
	Lig:C-Val163	Hydrophobic (Alkyl)	3.8819	
	Tyr62-Lig:C	Hydrophobic (Pi-Alkyl)	4.1690	
	Tyr62-Lig:C	Hydrophobic (Pi-Alkyl)	4.0323	
	His299-Lig:C	Hydrophobic (Pi-Alkyl)	4.7817	
Katononic acid	Lig:C-Trp59	Hydrophobic (Pi-Sigma)	3.7042	−8.6
	Val163-Lig	Hydrophobic (Alkyl)	4.4902	
	Val163-Lig	Hydrophobic (Alkyl)	4.2520	
	Lig:C-Val163	Hydrophobic (Alkyl)	3.4001	
	Lig:C-Leu165	Hydrophobic (Alkyl)	4.9166	
	Trp59-Lig	Hydrophobic (Pi-Alkyl)	5.4839	
	Trp59-Lig	Hydrophobic (Pi-Alkyl)	4.9116	
	Trp59-Lig:C	Hydrophobic (Pi-Alkyl)	4.1964	
	Trp59-Lig	Hydrophobic (Pi-Alkyl)	4.5411	
	Trp59-Lig:C	Hydrophobic (Pi-Alkyl)	4.7649	
	Trp59-Lig:C	Hydrophobic (Pi-Alkyl)	4.3555	
	Tyr62-Lig:C	Hydrophobic (Pi-Alkyl)	5.3466	
	Tyr62-Lig:C	Hydrophobic (Pi-Alkyl)	4.3793	
	His101-Lig:C	Hydrophobic (Pi-Alkyl)	4.7060	
	His305-Lig:C	Hydrophobic (Pi-Alkyl)	4.3501	
	His305-Lig:C	Hydrophobic (Pi-Alkyl)	4.8747	
**α-Glucosidase**				
3-oxolupenal	Arg608:HH21-Lig:O	Hydrogen Bond	2.4589	−7.9
	Glu866:HN-Lig:O	Hydrogen Bond	2.5890	
	Val867:HN-Lig:O	Hydrogen Bond	2.4786	
	Arg585-Lig	Hydrophobic (Alkyl)	5.2399	
	Val588-Lig	Hydrophobic (Alkyl)	4.7955	
	Leu865-Lig	Hydrophobic (Alkyl)	4.9869	
	Lig:C-Val588	Hydrophobic (Alkyl)	4.4625	
	Lig:C-Leu865	Hydrophobic (Alkyl)	3.6528	
	Lig:C-Arg594	Hydrophobic (Alkyl)	4.1884	
	Lig:C-Leu865	Hydrophobic (Alkyl)	4.8546	
	His717-Lig:C	Hydrophobic (Pi-Alkyl)	5.4121	
	His717-Lig:C	Hydrophobic (Pi-Alkyl)	4.1431	
Katononic acid	Glu866:HN-Lig:O	Hydrogen Bond	2.2888	−7.7
	Lys589:CE-Lig:O	Hydrogen Bond	3.6549	
	Val588-Lig	Hydrophobic (Alkyl)	4.9374	
	Lig:C-Arg585	Hydrophobic (Alkyl)	3.7005	
	Lig:C-Val588	Hydrophobic (Alkyl)	4.0007	
	Lig:C-Arg585	Hydrophobic (Alkyl)	4.2464	
	Lig:C-Arg594	Hydrophobic (Alkyl)	4.0545	
	Lig:C-Leu865	Hydrophobic (Alkyl)	5.0477	
	His584-Lig	Hydrophobic (Pi-Alkyl)	5.2947	
	His584-Lig:C	Hydrophobic (Pi-Alkyl)	4.2068	
	His584-Lig:C	Hydrophobic (Pi-Alkyl)	4.4082	
	His717-Lig	Hydrophobic (Pi-Alkyl)	5.1446	

## Supplementary Materials

The following are available online at https://www.mdpi.com/2218-273X/10/1/61/s1, Figure S1: GC-MS analysis of (A) 3-oxolupenal, and (B) katononic acid; Figure S2: NMR spectra of 3-oxolupenal; Figure S3: NMR spectra of katononic acid.
