# Supplementary material for: Alpha-Amylase and Alpha-Glucosidase Enzyme Inhibition and Antioxidant Potential of 3-Oxolupenal and Katononic Acid Isolated from Nuxia oppositifolia
Source: Biomolecules. 2019 Dec 30;10(1):61. doi: 10.3390/biom10010061 (PMC7022278; doi:10.3390/biom10010061)
Supplement: Supplementary file 1 [file biomolecules-10-00061-s001.pdf]

Supplementary data

Alpha-amylase and Alpha-glucosidase Enzyme Inhibition and Antioxidant Potential of 3-Oxolupenal and Katononic Acid Isolated from *Nuxia oppositifolia*

Ali S. Alqahtani[1,2], Syed Hidayathulla[1], Md Tabish Rehman[2*], Ali A. ElGamal[2], Shaza Al-Massarani[2], Valentina Razmovski-Naumovski[3], Mohammed S. Alqahtani[4], Rabab A. El Dib[5], Mohamed F. AlAjmi[2]

[1] Medicinal, Aromatic and Poisonous Plants Research Center (MAPRC), College of Pharmacy, King Saud University, PO Box 2457, Riyadh 11451, Saudi Arabia; alalqahtani@ksu.edu.sa (A.S.A.); hidayathsyed@gmail.com (S.H.)

[2] Department of Pharmacognosy, College of Pharmacy, King Saud University, PO Box 2457, Riyadh 11451, Saudi Arabia; aelgamel@ksu.edu.sa (A.A.E.); shazamas@yahoo.com (S.A.M.); malajmii@ksu.edu.sa (M.F.A.)

[3] South Western Sydney Clinical School, School of Medicine, University of New South Wales, Sydney, NSW 2052, Australia; v.naumovski@unsw.edu.au

[4] Department of Pharmaceutics, College of Pharmacy, King Saud University, Riyadh 11451, Saudi Arabia; msaalqahtani@ksu.edu.sa

[5] Department of Pharmacognosy, Faculty of Pharmacy, Helwan University, Cairo 11795, Egypt; reldib@yahoo.com

***Correspondence:** Email: mrehman@ksu.edu.sa; Tel.: +966-1467728

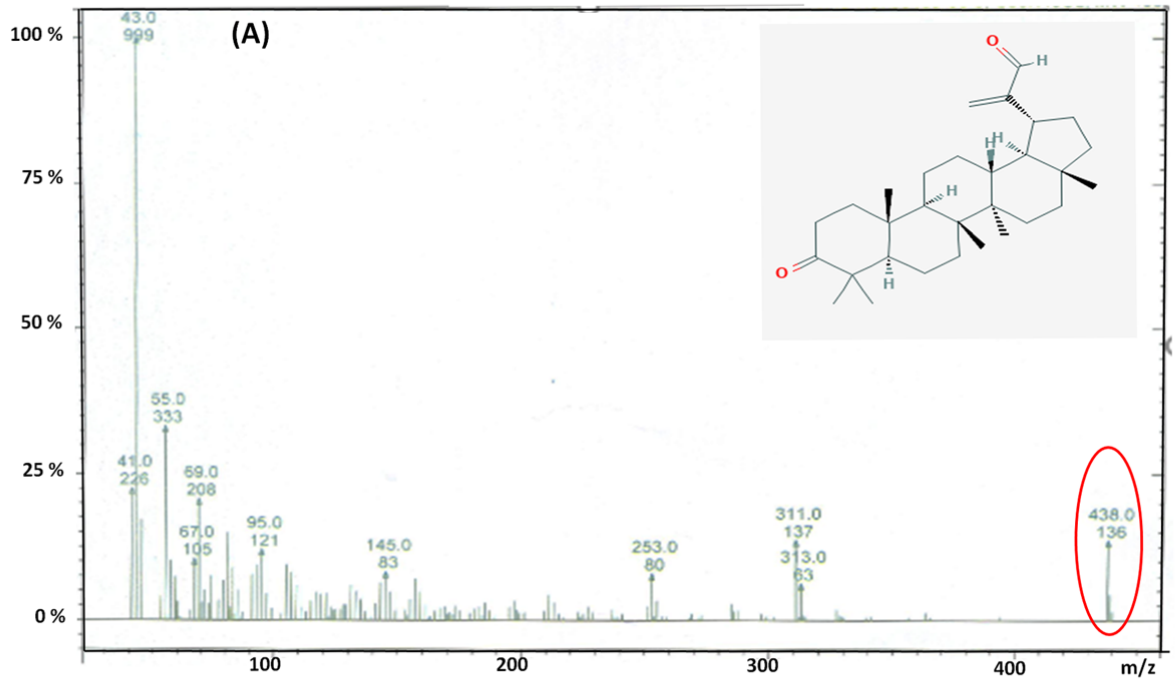

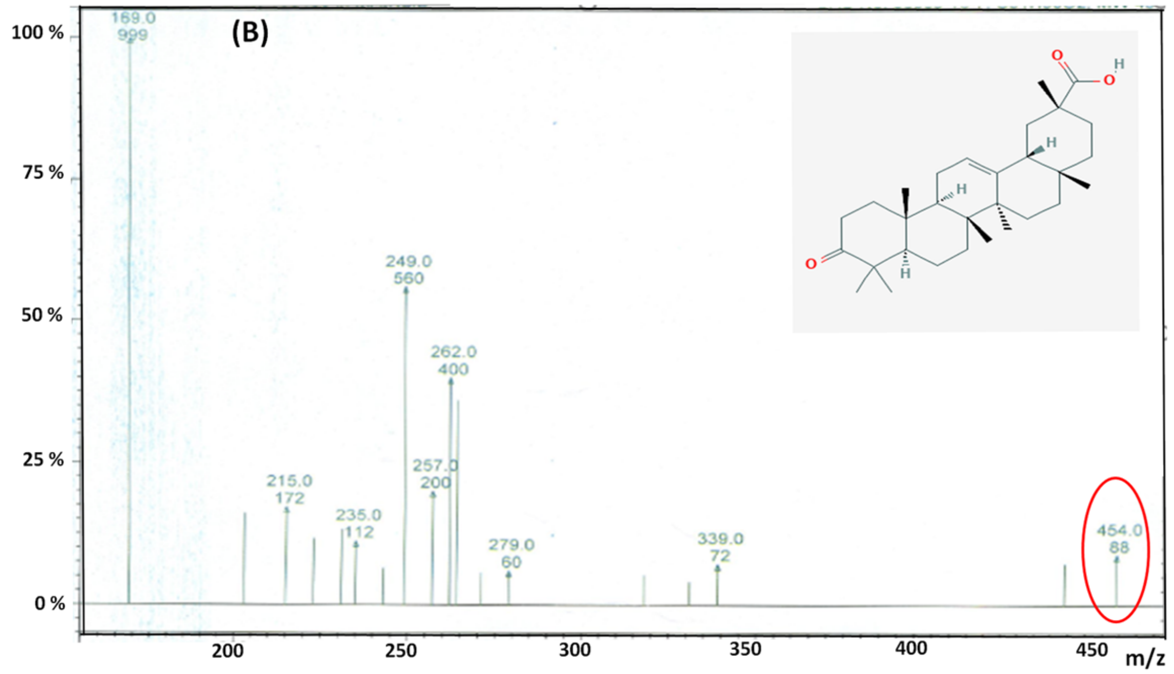

Figure S1. GC-MS analysis of (A) 3-oxolupenal (m/z = 438.0136 calculated for $C_{30}H_{46}O_2$, 438.3498), and (B) katononic acid (m/z 454.0880 calculated for $C_{30}H_{46}O_3$, 454.3603).

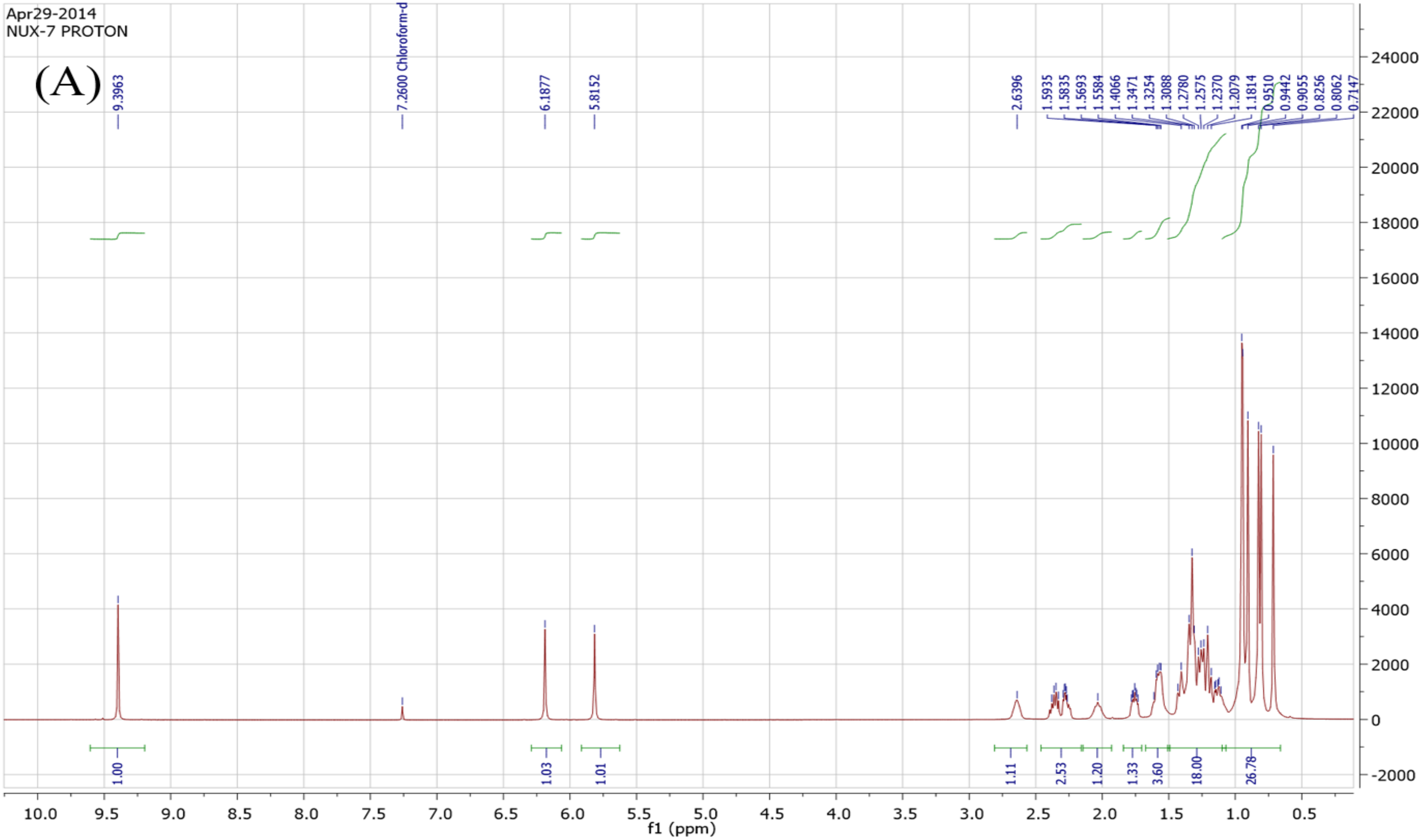
Apr29-2014
NUX-7 PROTON
(A)
9.3963
7.2600 Chloroform-d
6.1877
5.8152
2.6396
1.5935
1.5835
1.5693
1.5584
1.4066
1.3471
1.3254
1.3088
1.2780
1.2575
1.2370
1.2079
1.1814
0.9510
0.9442
0.9055
0.8256
0.8062
0.7147
1.00
1.03
1.01
1.11
2.53
1.20
1.33
3.60
18.00
26.78
f1 (ppm)

27

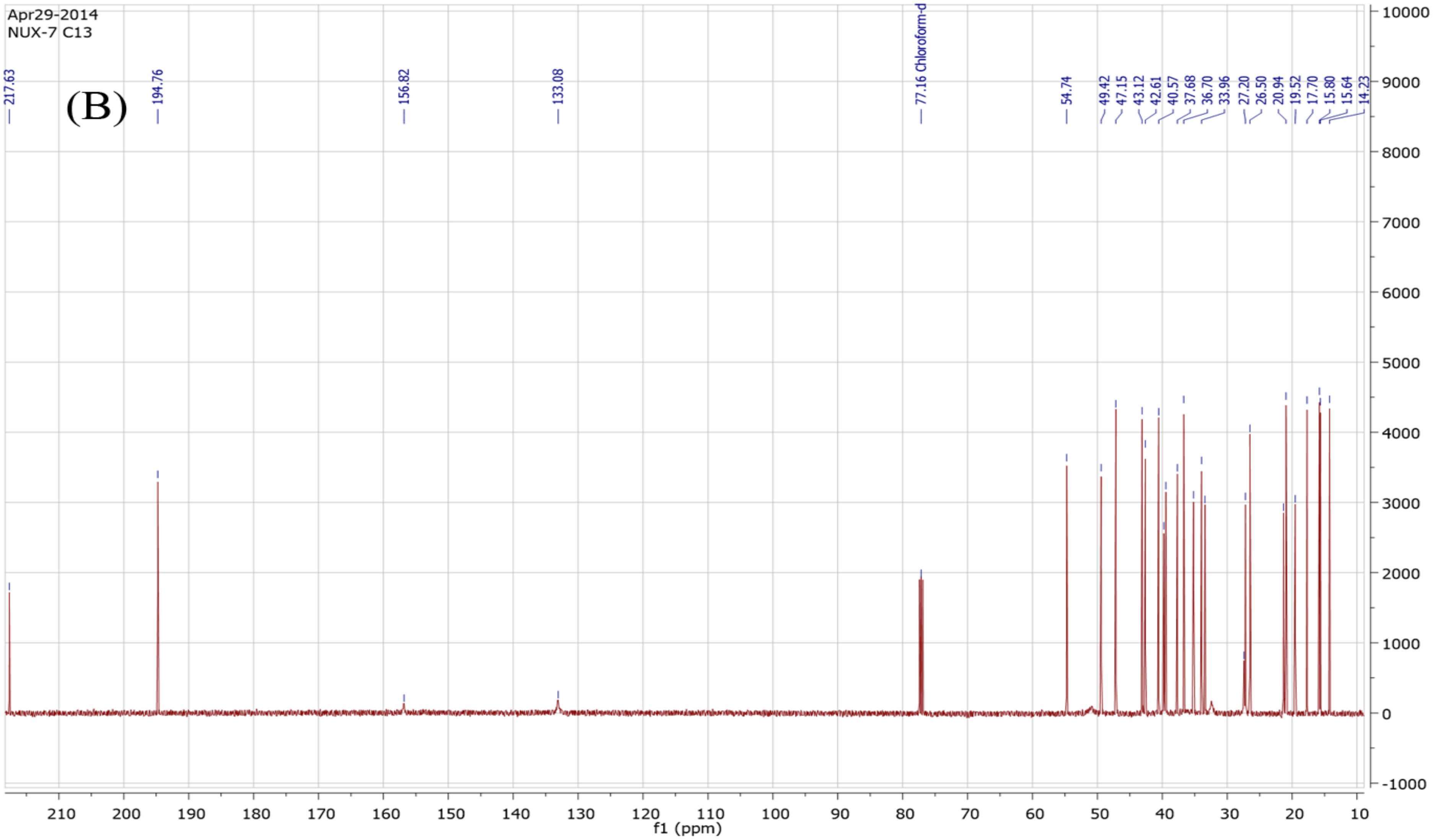
Apr29-2014
NUX-7 C13
(B)
217.63
194.76
156.82
133.08
77.16 Chloroform-d
54.74
49.42
47.15
43.12
42.61
40.57
37.68
36.70
33.96
27.20
26.50
20.94
19.52
17.70
15.80
15.64
14.23
f1 (ppm)

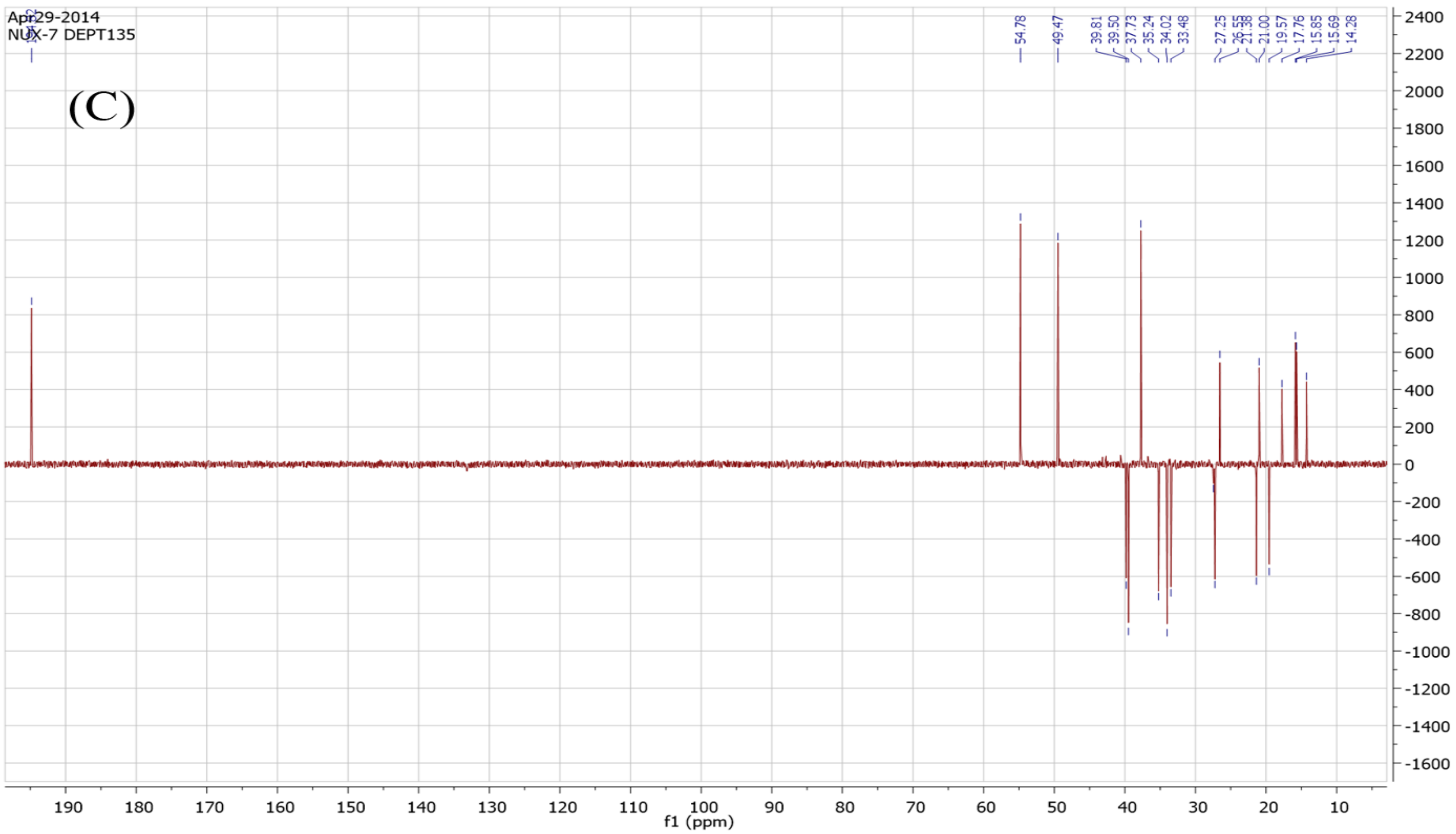

Figure S2. NMR spectra of 3-oxolupenal (A) ^{1}H, (B) ^{13}C, and (C) Depth. ^{1}H-NMR ($CDCl_3$, 500 MHz): δ 0.71 (s, Me-24), 0.80 (s, Me-28), 0.82 (s, Me-25), 0.90 (s, Me-27), 0.95 (s, Me-23), 1.18 (s, Me-26), 9.39 (s, H-30), 6.1,5.8 (s, H-29a.29b). ^{13}C-NMR($CDCl_3$, 125 MHz): δ 39.5(C-1), 34.0(C-2), 217.6(C-3), 47.2(C-4), 54.8(C-5), 19.6(C-6), 33.5(C-7), 40.6(C-8), 49.5(C-9), 36.7(C-10), 21.7(C-11), 27.5(C-12), 37.7(C-13), 42.7(C-14), 27.5(C-15), 35.2(C-16), 43.2(C-17), 51.0(C-18), 51.0(C-19), 157.2(C-20), 32.7(C-21), 39.8(C-22), 26.6(C-23), 21.0(C-24), 15.6(C-25), 16.0(C-26), 14.7(C-27), 17.8(C-28), 133.2(C-29), 194.8(C-30).

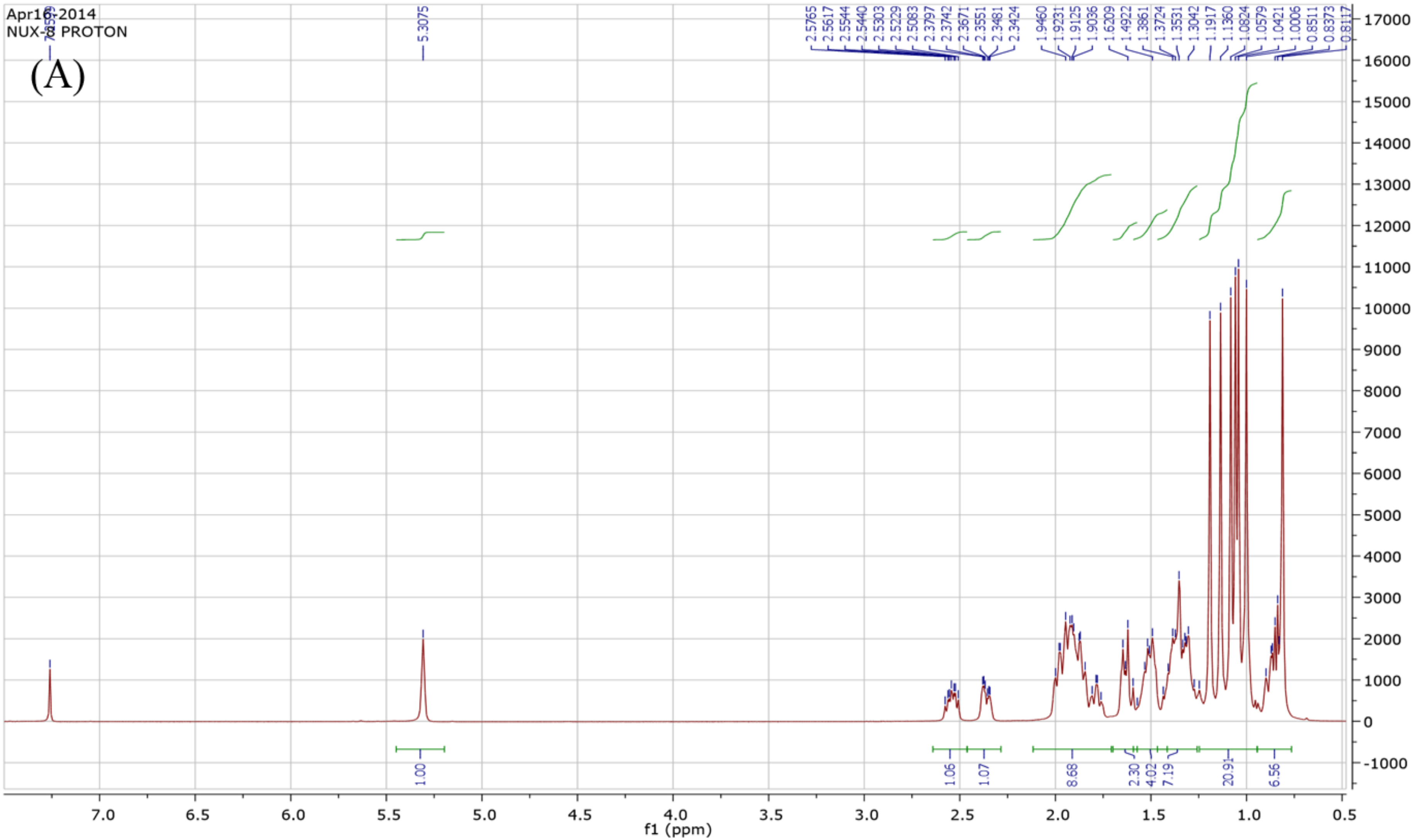
Apr16-2014
NUX-8 PROTON
(A)
5.3075
1.00
1.06
1.07
8.68
2.30
4.02
7.19
20.91
6.56
f1 (ppm)

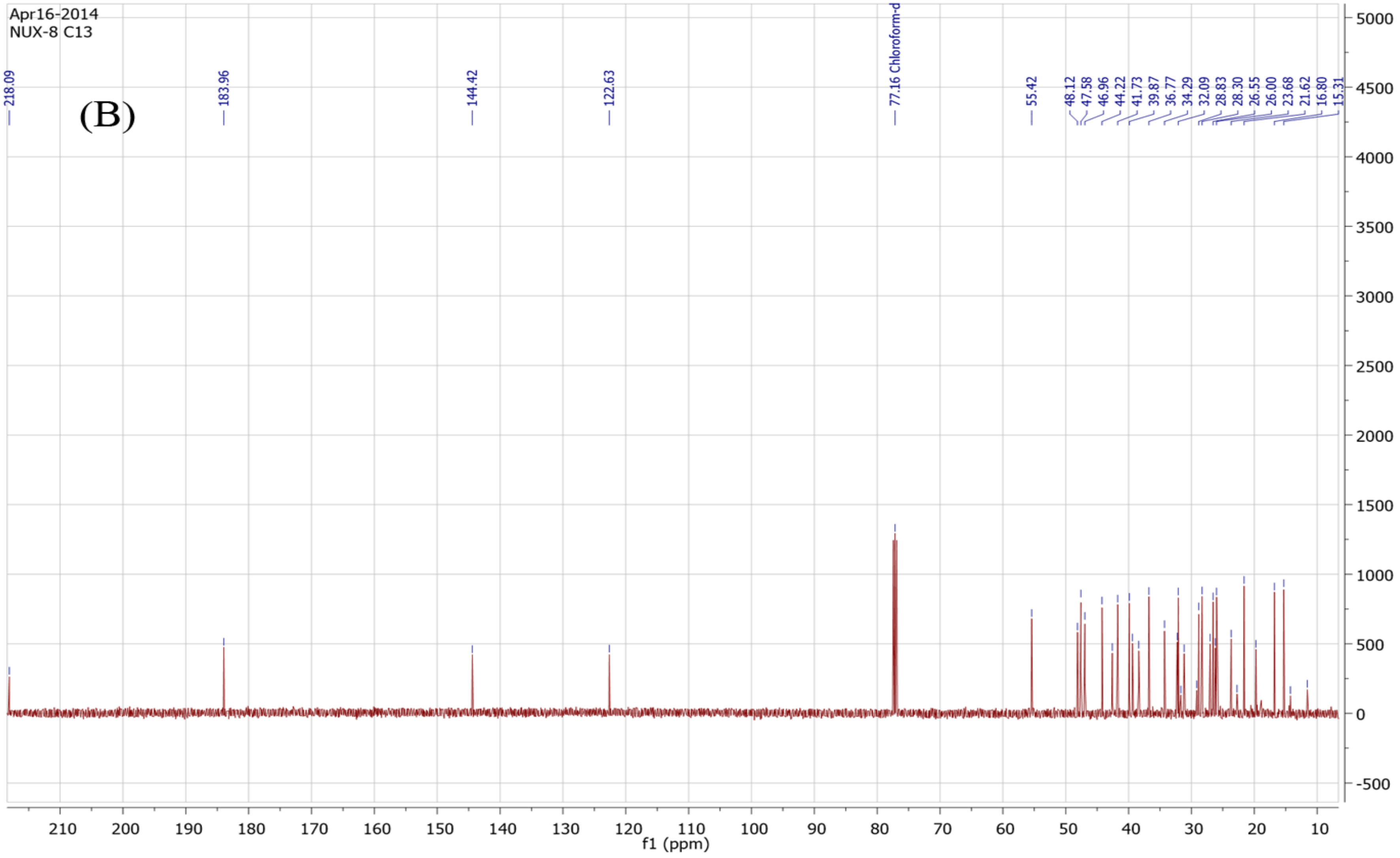
Apr16-2014
NUX-8 C13
(B)
218.09
183.96
144.42
122.63
77.16 Chloroform-d
55.42
48.12
47.58
46.96
44.22
41.73
39.87
36.77
34.29
32.09
28.83
28.30
26.55
26.00
23.68
21.62
16.80
15.31
f1 (ppm)

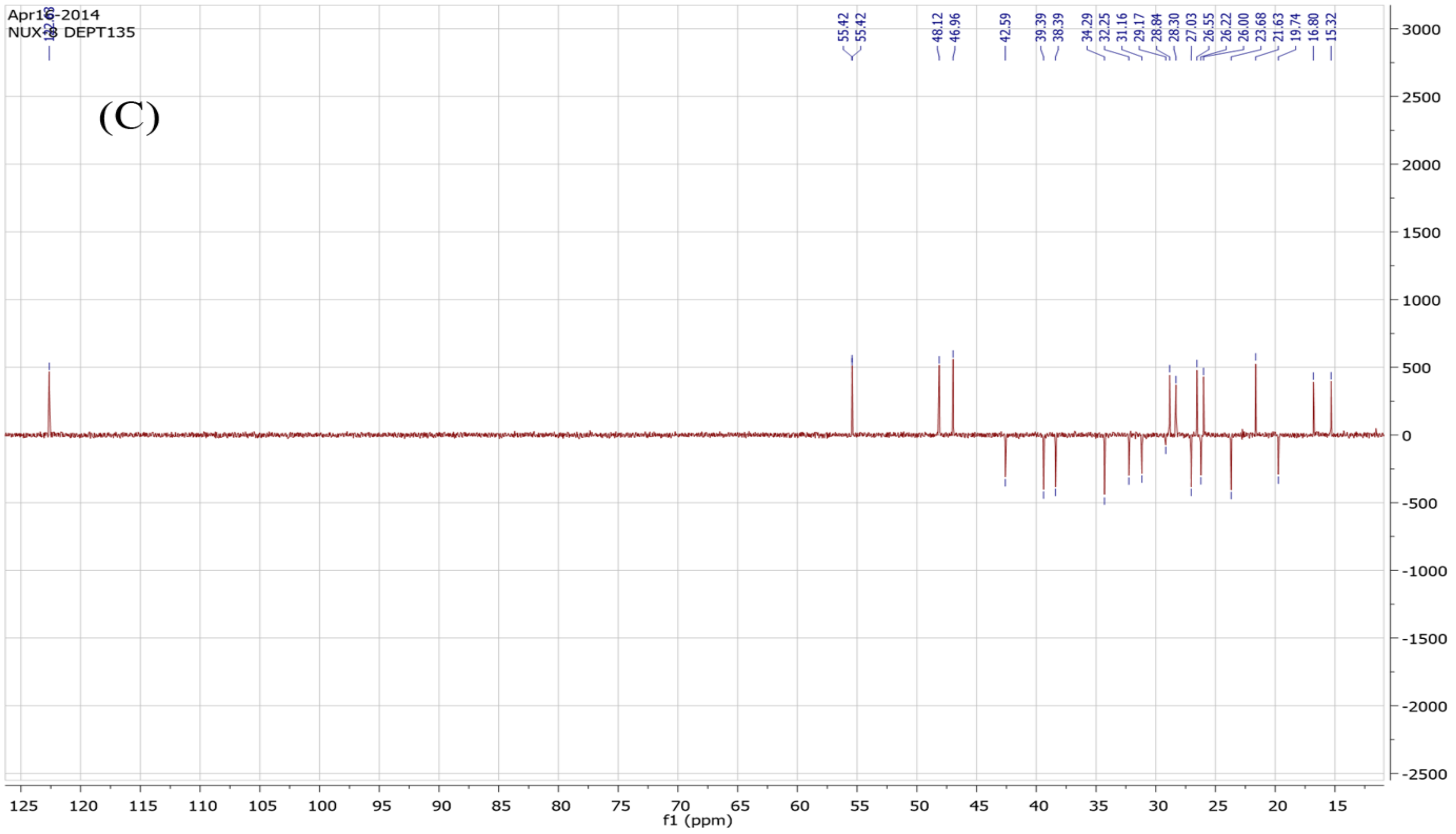

Figure S3. NMR spectra of katononic acid (A) ^{1}H, (B) ^{13}C, and (C) Depth. ^{1}H-NMR ($CDCl_3$, 500 MHz): δ 0.81 (s, Me-24), 0.83 (s, Me-28), 0.85 (s, Me-25), 1.00 (s, Me-27), 1.04 (s, Me-23), 1.13 (s, Me-26), 1.19 (s H-29), 5.30 (s, H-12). ^{13}C-NMR ($CDCl_3$, 125 MHz): δ 39.3(C-1), 34.2(C-2), 217.6(C-3), 47.5(C-4), 55.3(C-5), 19.6(C-6), 32.2(C- 7), 39.7(C-8), 48.0(C-9), 36.7(C-10), 23.6(C-11), 122.5(C-12), 144.3(C-13), 41.6(C-14), 26.1(C-15), 26.9(C-16), 32.0(C-17), 47.0(C-18), 42.5(C-19), 44.1(C-20), 31.1(C-21), 38.3(C-22), 26.4(C-23), 21.5(C-24), 15.2(C-25), 16.7(C-26), 25.9(C-27), 28.2(C-28), 28.7(C-29), 183.9(C-30).

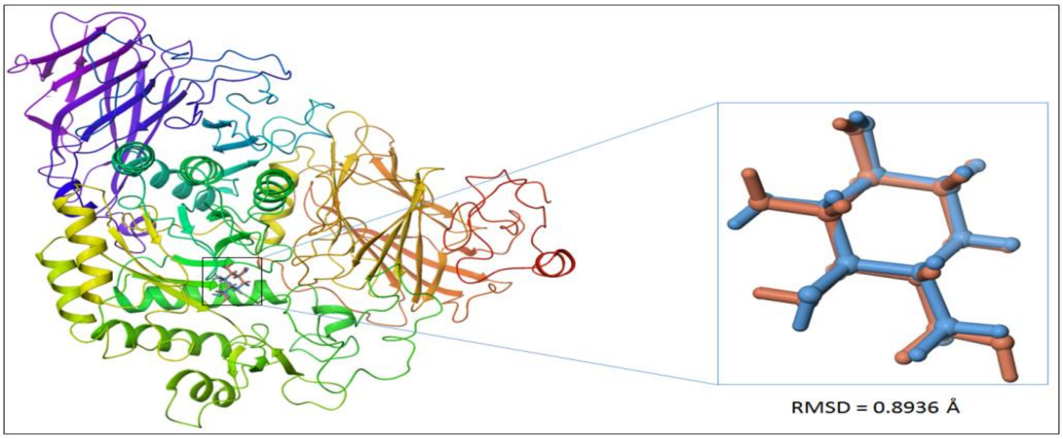

(A)

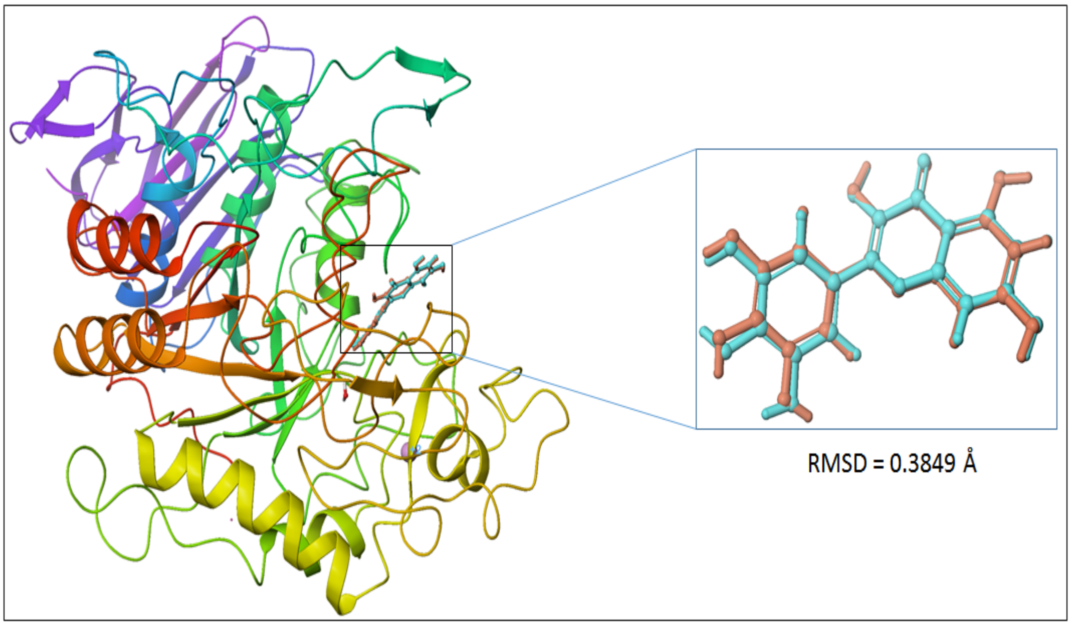

(B)

Figure S4. Validation of molecular docking protocol by re-docking (A) myricetin at the active site of α-amylase, and (B) 1-deoxynojirimycin at the active site of α-glucosidase.
